# Lorazepam and Survival in Asian Patients with Pancreatic Cancer: A Retrospective Cohort Study

**DOI:** 10.1007/s12029-026-01429-7

**Published:** 2026-03-02

**Authors:** Tae Seung Lee, Jin Ho Choi, In Rae Cho, Jin Woo Park, Sang Hyub Lee, Ji Kon Ryu, Woo Hyun Paik

**Affiliations:** 1 Division of Gastroenterology, Department of Internal Medicine, Seoul National University Hospital, Daehak-Ro, Jongno-Gu, 101 Seoul, Republic of Korea; 2 Department of Pathology, Yonsei University College of Medicine, Seoul, Republic of Korea

**Keywords:** Lorazepam, Pancreatic neoplasm, Chemotherapy, Antiemetics, Benzodiazepine

## Abstract

**Purpose:**

Lorazepam is frequently used in pancreatic cancer patients receiving chemotherapy for its antiemetic properties and to mitigate psychological distress. However, N-unsubstituted benzodiazepines like lorazepam may adversely impact pancreatic cancer progression by stimulating fibrosis and inflammatory signaling. This study aimed to retrospectively compare the survival rates of pancreatic cancer patients exposed to lorazepam with those who were not exposed to real-world clinical practice.

**Methods:**

Data were retrospectively reviewed from patients aged ≥ 18 years with pathologically confirmed pancreatic cancer who received palliative chemotherapy at Seoul National University Hospital between January 2011 and January 2023. Patients were dichotomized based on lorazepam administration: those who received ≥ 15 mg (equivalent to ≥ 1 tablet daily for 30 days) were classified as the high-dose group, and those who received < 15 mg were classified as the low-dose group. The relationship between lorazepam exposure and progression-free survival (PFS), as well as overall survival (OS), was analyzed.

**Results:**

Among pancreatic cancer patients undergoing palliative chemotherapy, PFS was worse in the high-dose lorazepam group compared to the low-dose group [median, 6 months (5–7) vs. 8 months (7–9), p = 0.025]. However, there was no difference in OS between the two groups [median, 11 months (10–12) vs. 12 months (11–13), p = 0.465].

**Conclusion:**

Higher cumulative lorazepam exposure was associated with shorter progression-free survival in Asian patients with pancreatic cancer treated with palliative chemotherapy.

**Supplementary Information:**

The online version contains supplementary material available at 10.1007/s12029-026-01429-7.

## Introduction

Pancreatic cancer is one of the most challenging cancers to treat with poor prognosis. Surgical resection is the only curative method, however, fewer than 20% of patients are eligible for surgery at the time of diagnosis, resulting in a five-year survival rate of about 13% [1]. This low eligibility is due to the aggressive nature of pancreatic cancer, which often progresses locally or metastasizes systemically in its early stages. At the time of diagnosis, about 50%–60% of patients are found to have metastatic disease [2], 25%–30% have locally advanced disease [2], and only 10%–15% are diagnosed with localized disease suitable for surgery [2]. Even after surgery, recurrence is common [2]. Furthermore, the incidence of pancreatic cancer is increasing globally, with an annual rise of 0.5%–1% observed in the United States and South Korea. By 2030, pancreatic cancer is projected to become the second leading cause of cancer-related deaths [3]. Despite advancements in anti-cancer treatments [4–7], pancreatic cancer still has a low five-year survival rate. This grave prognosis is largely attributed to the tumor microenvironment, which is characterized by the presence of cancer-associated fibroblasts (CAFs) [8]. These CAFs contribute to treatment resistance by fostering a desmoplastic and fibrotic environment that diminishes the efficacy of chemotherapy [9, 10].

Given these huddles, recent studies have emphasized the importance of palliative care in pancreatic cancer, highlighting its impact on both treatment management and patient outcomes [11]. Pancreatic cancer’s high recurrence rate leads to significant emotional distress, anxiety, and depression among patients [12–14]. From the perspective of comprehensive cancer care, psychosocial support has become increasingly recognized as an essential aspect of patient management. In particular, anxiety and depression induced by cancer-related symptoms and pain severely affect patients’ quality of life [12]. Additionally, anticipatory anxiety and chemotherapy-induced nausea can reduce treatment compliance, thereby worsening quality of life, and performance status, and treatment outcomes [15].

To cope with these cancer-related effects, patients are often prescribed various palliative care medications, such as selective serotonin reuptake inhibitors, opioids, and benzodiazepines. However, increasing evidence suggests that many of these medications may influence cancer risk, tumor progression [16], and the efficacy of chemotherapy [17], either positively or negatively. Specifically, lorazepam is widely used not only to alleviate psychological distress during chemotherapy but also as an antiemetic to manage anticipatory nausea [18, 19].

Recent study has revealed that N-unsubstituted benzodiazepines, including lorazepam, clonazepam, nordiazepam, and oxazepam, promote the expression of GPR68, a receptor commonly found in the CAFs of pancreatic cancer [11]. This receptor increases IL-6 production in CAFs, which activates fibrotic and inflammatory signaling pathways. These pathways promotes desmoplasia and ischemic necrosis, ultimately correlated with poor progression-free survival (PFS) in pancreatic cancer patients [11]. Conversely, N-substituted benzodiazepines, such as alprazolam, diazepam, and temazepam, do not stimulate GPR68 activation [11].

In addition to these mechanistic findings, a recent clinical study by Tsilimigras et al. [16] evaluated the association between long-term lorazepam use and survival outcomes in patients with pancreatic adenocarcinoma. The authors reported that prolonged lorazepam exposure was associated with worse progression-free survival, while no significant difference in overall survival was observed. Although this study provided important clinical insight, it was conducted predominantly in non-Asian populations and did not specifically evaluate cumulative dose-based stratification.

Nonetheless, clinical data on the impact of lorazepam on the prognosis of pancreatic cancer patients remain limited. Therefore, this study aims to retrospectively evaluate the prognosis of pancreatic cancer patients receiving chemotherapy, comparing outcomes based on lorazepam use, which is prescribed to alleviate psychological distress and manage anticipatory nausea.

## Materials & Methods

### Study Population

Patients aged ≥ 18 years with pathologically confirmed pancreatic cancer who received palliative chemotherapy at Seoul National University Hospital from January 2011 to January 2023, were enrolled in this study. All included patients were of Asian ethnicity, reflecting the single-race population of this single-center study conducted in Korea. As this is a retrospective study, informed consent was not obtained from the patients. The retrospective review included patients regardless of whether they underwent surgery or radiation therapy. All prescriptions for alprazolam and lorazepam up to the last follow-up date were reviewed, regardless of the cancer diagnosis. Patients who had been prescribed alprazolam, or both lorazepam and alprazolam, even once, were excluded from the study.

Patients were categorized into two groups based on cumulative lorazepam exposure, defined as the total prescribed dose aggregated across multiple prescription periods. In a previous study [16], outcomes were compared between patients exposed to lorazepam for ≥ 30 days and those with shorter exposure. Based on this concept, cumulative lorazepam dose was operationalized using a standard daily dose of 0.5 mg, corresponding to a 30-day exposure (15 mg). Accordingly, patients with a cumulative dose of < 15 mg (as a monthly total dose) were classified as the low-dose group, whereas those with ≥ 15 mg were classified as the high-dose group. Clinical and demographic parameters were retrospectively collected from electronic medical records, including age, sex, date of the initial imaging diagnosis of pancreatic cancer, tumor location and size, date of surgical resection, the initial and final dates of palliative chemotherapy, and the type of chemotherapy administered.

### Study Outcomes

The main endpoints were progression-free survival (PFS) and overall survival (OS). Both PFS and OS were measured from the initiation date of first-line palliative chemotherapy. Disease progression was determined by the first radiological identification of a new lesion or recurrence. Death was recorded based on documented evidence, or if unavailable, verified using mortality data. For patients classified as hopeless discharge or lost to follow-up under critical conditions, the last recorded follow-up date was regarded as the date of death.

### Statistical Methods

Qualitative variables were compared using the chi-square test or Fisher’s exact test, while quantitative variables underwent a normality assessment via the Shapiro–Wilk test. When analyzing continuous variables, the Student’s t-test was applied for normally distributed data, whereas the Mann–Whitney U-test was used for non-normal distributions. Categorical variables were examined with either the chi-square test or Fisher’s exact test. A p-value of ≤ 0.05 was considered statistically significant. All statistical tests were performed as two-sided tests, and relevant variables were included as covariates in further analyses.

For PFS (progression-free survival) and OS (overall survival), 95% confidence intervals (CIs) were estimated using the Kaplan–Meier method and reported as the median and range. The incidence of adverse events was expressed in percentages, with differences assessed via the chi-square test, applying a 5% significance level. Additionally, a multivariable Cox proportional hazards model was utilized to evaluate factors deemed clinically significant in their influence on PFS and OS.

### Statement of Human and Animal Rights

This study was approved by the Institutional Review Board of Seoul National University hospital.

## Results

### Clinical Characteristics of the study population

**Fig. 1:**
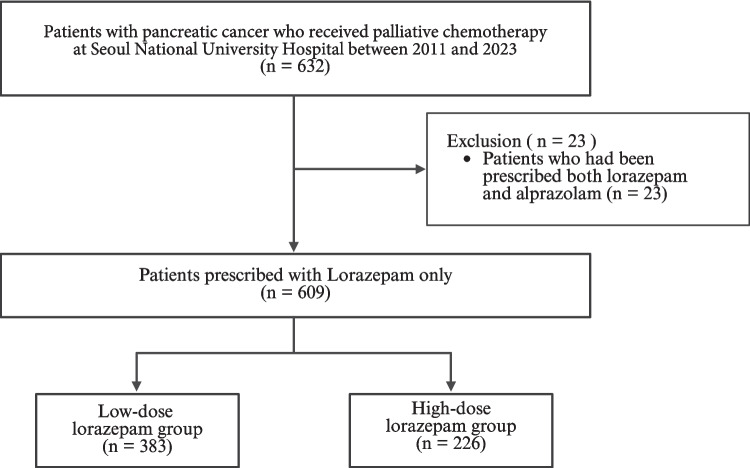
Flow diagram of the study

Of the 632 patients, 23 who had been prescribed both lorazepam and alprazolam were excluded. The remaining 383 patients who were exposed to less than 15 mg of lorazepam were categorized as the low-dose group, while the 226 patients who were exposed to more than 15 mg of lorazepam were categorized as the high-dose group (Fig. 1).

In the baseline characteristics, the high-dose group had a statistically significantly higher proportion of younger patients (p = 0.037). However, a significantly greater percentage of patients in the high-dose group presented with unresectable disease at diagnosis compared to the low-dose group (81.9% vs. 89.8%, p = 0.012). Additionally, the low-dose group had a higher proportion of patients who underwent curative resection prior to chemotherapy (22.2% vs. 11.9%, p = 0.002). There were no differences between the two groups in terms of sex, tumor size, tumor location, conversion to resection, number of palliative chemotherapy line, palliative chemotherapy duration, no evidence of disease status, or follow-up duration (Table 1). Detailed information on palliative chemotherapy regimens by treatment line is provided in Supplementary Table 1.

**Table 1:** Baseline characteristics of the study patients^*^

	Low-dose group	High-dose group	p-value
	(N = 383)	(N = 226)	
Sex			0.288
Male	240 (62.7%)	131 (58.0%)	
Female	143 (37.3%)	95 (42.0%)	
Age	62.0 ± 9.1	60.2 ± 10.6	0.037
Highest grade of chemotherapy related nausea/vomiting			< 0.001
No Adverse event	40 (10.4%)	11 (4.9%)	
Grade 1	73 (19.1%)	29 (12.8%)	
Grade 2	30 (7.8%)	9 (4.0%)	
Grade 3	180 (47.0%)	119 (52.7%)	
Grade 4	60 (15.7%)	58 (25.7%)	
Lorazepam prescription dose (mg)	1.8 ± 3.6	185.4 ± 487.2	< 0.001
Lorazepam prescription days	0.9 ± 2.9	91.2 ± 488.2	0.006
Tumor size (centimeter)	3.7 ± 1.7	3.9 ± 1.9	0.189
Tumor location			0.155
Head	165 (43.1%)	82 (36.3%)	
Body	102 (26.6%)	75 (33.2%)	
Tail	116 (30.3%)	69 (30.5%)	
Unresectable at diagnosis	313 (81.9%)	203 (89.8%)	0.012
Curative resection before chemotherapy	85 (22.2%)	27 (11.9%)	0.002
Conversion to resection	20 (5.2%)	11 (4.9%)	0.999
Line of palliative chemotherapy	2.2 ± 1.0	2.2 ± 0.9	0.965
Progression	323 (84.3%)	199 (88.1%)	0.251
Death	372 (97.1%)	221 (97.8%)	0.818
NED^†^ state	3 (0.8%)	2 (0.9%)	1.000
Palliative chemotherapy duration (months)	12.9 ± 14.7	11.6 ± 10.7	0.211
Follow up duration (months)	15.6 ± 18.1	14.8 ± 16.2	0.608

^*^ Values are presented as mean ± standard deviation. Other values are presented as number (%)

^†^*NED*, No evidence of disease

### Primary and Secondary Endpoints

**Fig. 2:**
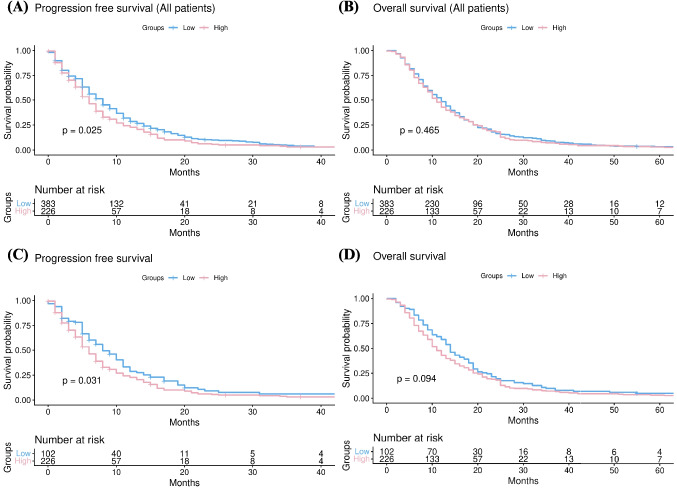
Comparison of (**a**) progression-free survival and (**b**) overall survival between high-dose and low-dose lorazepam groups in all patients, and (**c**) progression-free survival and (d) overall survival among patients treated with lorazepam

PFS was worse in the high-dose lorazepam group compared to the low-dose group [median, 6 months (5–7) vs. 8 months (7–9), p = 0.025] (Fig. 2A). However, there was no difference in OS between low-dose group and high-dose group [median, 11 months (10–12) vs. 12 months (11–13), p = 0.465] (Fig. 2B). Given the potential heterogeneity introduced by including patients with no lorazepam exposure in the low-dose group, we performed an additional subgroup analysis excluding these patients. This approach allowed us to specifically evaluate the dose–response relationship among patients who received lorazepam. In this subgroup, PFS remained significantly worse in the high-dose lorazepam group compared to the low-dose group [median, 6 months (5–7) vs. 8 months (7–11), p = 0.031] (Fig. 2C). However, there was no difference in OS between low-dose group and high-dose group [median, 11 months (10–12) vs. 14 months (13–17), p = 0.094] (Fig. 2D).

### Covariate-Adjusted Analysis of PFS and OS Based on Lorazepam Usage

In the univariable Cox regression analysis for PFS, lorazepam dosage and tumor location (head) were associated with a higher hazard ratio (HR), indicating worse outcomes. In contrast, highest nausea/vomiting grade and conversion to curative resection after chemotherapy were associated with a lower HR, suggesting improved outcomes. For OS, older age and female was associated with a higher HR, whereas highest nausea/vomiting grade, whether curative surgery was performed before chemotherapy, conversion to curative resection after chemotherapy, and the number of palliative chemotherapy lines were associated with a lower HR (Table 2).

**Table 2:** Univariable Cox regression analysis for (A) Progression-free survival (B) Overall survival

	PFS		OS	
	Hazard Ratio (95% Cl)	p-value	Hazard Ratio (95% Cl)	p-value
Lorazepam dosage (High vs. Low)	1.23 (1.03–1.47)	0.023	1.06 (0.90–1.26)	0.462
Age (per year)	1.00 (1.00–1.01)	0.363	1.01 (1.00–1.02)	0.006
Sex (Female vs. Male)	0.92 (0.77–1.09)	0.339	0.84 (0.71–1.00)	0.045
Highest nausea/vomiting grade (per grade)	0.92 (0.86–0.99)	0.030	0.83 (0.78–0.89)	< 0.001
Tumor size (Per centimeter)	1.01 (0.97–1.06)	0.616	1.04 (1.00–1.09)	0.069
Tumor location (Head vs. body/tail)	1.24 (1.04–1.48)	0.017	1.17 (1.00–1.39)	0.055
Unresectable at diagnosis (Yes vs. No)	1.05 (0.83–1.34)	0.661	1.16 (0.92–1.46)	0.205
Pre-chemotherapy resection (Yes vs. No)	0.95 (0.76–1.18)	0.620	0.73 (0.59–0.91)	0.004
Conversion to resection (Yes vs. No)	0.33 (0.22–0.50)	< 0.001	0.24 (0.16–0.37)	< 0.001
First Line Chemotherapy (Gemcitabine/Nab-Paclitaxel vs. FOLFIRINOX)	1.11 (0.92–1.35)	0.271	1.11 (0.32–1.32)	0.245
Total line of chemotherapy (Per the number of line)	1.01 (0.94–1.09)	0.759	0.74 (0.69–0.80)	< 0.001

**Fig. 3:**
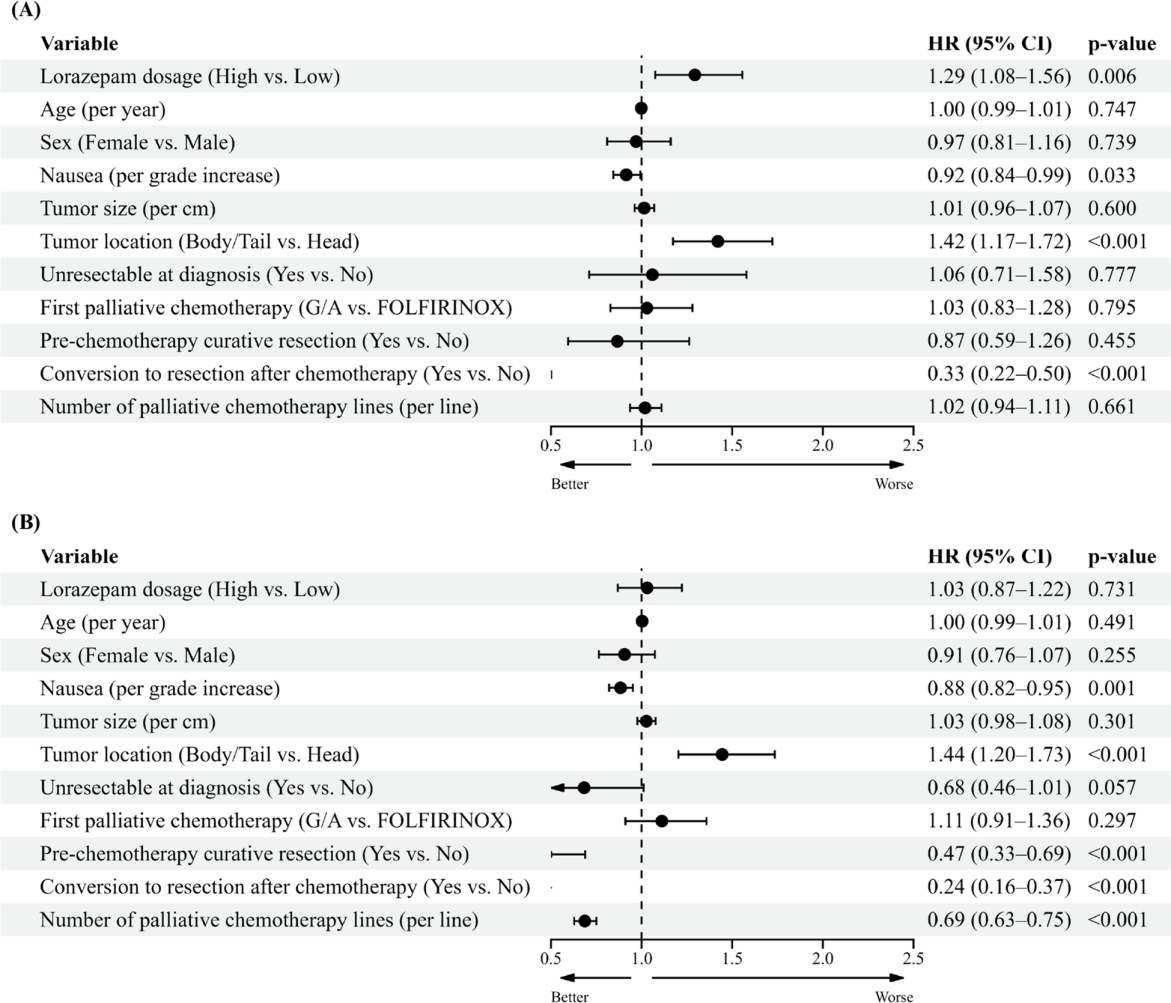
Covariate − adjusted analysis of (**a**) Progression free survival and (**b**) Overall survival based on Lorazepam dosage

A multivariable Cox regression analysis was performed, adjusting for factors such as lorazepam dosage, age, sex, highest nausea/vomiting grade, tumor size, tumor location, unresectable status at diagnosis, first chemotherapy regimen, whether curative surgery was performed before chemotherapy, conversion to surgery after chemotherapy, and the number of palliative chemotherapy lines. High-dose lorazepam was associated with a significantly higher HR for PFS [HR 1.29 (95% CI, 1.08–1.56), p = 0.006]. Also, head location of tumor [HR 1.42 (95% CI, 1.17–1.72), p < 0.001] was associated with a significantly higher HR for PFS. Conversely, the higher nausea grade [HR 0.92 (95% CI, 0.84–0.99), p = 0.033] and conversion to resection [HR 0.33 (95% CI, 0.22–0.50), p < 0.001], PFS was found to have a significantly lower HR (Fig. 3A).For OS, no significant difference was observed based on lorazepam dosage [HR 1.02 (95% CI, 0.86–1.21), p = 0.874]. In cases with head location of tumor [HR 1.45 (95% CI, 1.21–1.74), p < 0.001] was associated with a significantly higher HR for PFS. Conversely, the higher nausea grade [HR 0.88 (95% CI, 0.82–0.95), p = 0.001], whether curative surgery was performed before chemotherapy [HR 0.47 (95% CI, 0.33–0.69), p < 0.001], conversion to resection [HR 0.24 (95% CI, 0.16–0.37), p < 0.001], and the number of palliative chemotherapy [HR 0.69 (95% CI, 0.63–0.75), p < 0.001], OS was found to have a significantly lower HR (Fig. 3B).

## Discussion

Lorazepam is frequently used in clinical practice to manage chemotherapy-related anticipatory nausea and vomiting [18, 19]. Previous studies have demonstrated that lorazepam can promote the progression of pancreatic cancer by inducing IL-6 secretion from CAFs [11]. Additionally, a prior research suggested that long-term use of lorazepam might be associated with a worse prognosis in surgically resected pancreatic cancer patients [16]. However, This study included only 55%–60% of patients who received chemotherapy [16], a lower proportion compared to our study, which specifically compares survival outcomes based on lorazepam use in patients who received palliative chemotherapy.

Although data on ethnic differences in lorazepam metabolism are limited, benzodiazepines as a drug class are metabolized by hepatic enzymes that exhibit genetic polymorphisms [20]. Variations in the frequency of these polymorphic drug-metabolizing enzymes across racial and ethnic groups may lead to differences in drug exposure, duration of action, and clinical effects. In our study, similar to previous reports [11, 16], patients with a relatively higher cumulative dose of lorazepam tended to have shorter PFS compared with those in the lower cumulative dose group. Interestingly, no significant difference in OS was observed. This finding *may* in part reflect the homogeneous ethnic composition of the study population, as all patients were of Asian ethnicity, in whom differences in drug metabolism could potentially influence clinical outcomes. In addition, among patients receiving palliative chemotherapy, lorazepam might play a supportive role by alleviating anticipatory nausea and reducing psychological stress, which could help maintain chemotherapy adherence and allow prolonged treatment duration. Such effects might partially explain the absence of a significant difference in OS between groups [21].

Our study has several limitations: 1) Since the investigation was conducted retrospectively by reviewing prescription records, it does not fully reflect the actual intake of lorazepam. 2) The number of patients prescribed alprazolam alone was not sufficient for comparison 3) Although all patients received palliative chemotherapy, detailed information on the exact timing of lorazepam initiation relative to chemotherapy was not available, precluding time-dependent analyses. Therefore, the observed association between lorazepam exposure and progression-free survival should not be interpreted as causal, and residual confounding by symptom burden or treatment-related factors cannot be excluded.

In conclusion, higher cumulative lorazepam exposure was associated with shorter progression-free survival in Asian patients with pancreatic ductal adenocarcinoma receiving palliative chemotherapy. Given the retrospective design and lack of time-dependent exposure data, these findings should not be interpreted as causal. Further prospective studies are warranted to clarify the clinical implications of lorazepam use in this population.

## Supplementary Information

Below is the link to the electronic supplementary material.

Supplementary file1 (DOCX 53 KB)

## Data Availability

The data supporting the findings of this study are available from the corresponding author, Woo Hyun Paik, upon reasonable request. De-identified patient-level data will be shared in compliance with institutional and ethical regulations.

## Abbreviations

PFS: Progression-Free Survival
OS: Overall Survival
CAFs: Cancer-Associated Fibroblasts
HR: Hazard Ratio
CI: Confidence Interval
GPR68: G Protein-Coupled Receptor 68

## References

[1] Siegel RL, Giaquinto AN, Jemal A. Cancer statistics, 2024. CA Cancer J Clin. 2024;74:12–49. 10.3322/caac.21820. 38230766 10.3322/caac.21820

[2] Kolbeinsson HM, Chandana S, Wright GP, Chung M. Pancreatic cancer: a review of current treatment and novel therapies. J Invest Surg. 2023;36:2129884. 10.1080/08941939.2022.2129884. 36191926 10.1080/08941939.2022.2129884

[3] Siegel RL, Miller KD, Jemal A. Cancer statistics, 2019. CA Cancer J Clin. 2019;69:7–34. 10.3322/caac.21551. 30620402 10.3322/caac.21551

[4] Wang-Gillam A, Li C-P, Bodoky G, et al. Nanoliposomal irinotecan with fluorouracil and folinic acid in metastatic pancreatic cancer after previous gemcitabine-based therapy (NAPOLI-1): a global, randomised, open-label, phase 3 trial. Lancet. 2016;387:545–57. 10.1016/S0140-6736(15)00986-1. 26615328 10.1016/S0140-6736(15)00986-1

[5] Von Hoff DD, Ervin T, Arena FP, et al. Increased survival in pancreatic cancer with nab-paclitaxel plus gemcitabine. N Engl J Med. 2013;369:1691–703. 10.1056/NEJMoa1304369. 24131140 10.1056/NEJMoa1304369 PMC4631139

[6] Raphael MJ, Raskin W, Habbous S, et al. The association of drug-funding reimbursement with survival outcomes and use of new systemic therapies among patients with advanced pancreatic cancer. JAMA Netw Open. 2021;4:e2133388. 10.1001/jamanetworkopen.2021.33388. 34779846 10.1001/jamanetworkopen.2021.33388 PMC8593760

[7] Conroy T, Desseigne F, Ychou M, et al. FOLFIRINOX versus gemcitabine for metastatic pancreatic cancer. N Engl J Med. 2011;364:1817–25. 10.1056/NEJMoa1011923. 21561347 10.1056/NEJMoa1011923

[8] Lee TS. Are probiotics beneficial or harmful for pancreatic cancer outcomes? Probiotics & Antimicro Prot. 2024. 10.1007/s12602-024-10437-7. 10.1007/s12602-024-10437-7 PMC12405297 39714574

[9] Golan T, Khvalevsky EZ, Hubert A, et al. Rnai therapy targeting KRAS in combination with chemotherapy for locally advanced pancreatic cancer patients. Oncotarget. 2015;6:24560–70. 10.18632/oncotarget.4183. 26009994 10.18632/oncotarget.4183 PMC4695206

[10] Lee TS, Kim JY, Lee MH, et al. Savolitinib: a promising targeting agent for cancer. Cancers. 2023;15:4708. 37835402 10.3390/cancers15194708 PMC10571651

[11] Cornwell AC, Tisdale AA, Venkat S, et al. Lorazepam stimulates IL6 production and is associated with poor survival outcomes in pancreatic cancer. Clin Cancer Res. 2023;29:3793–812. 10.1158/1078-0432.Ccr-23-0547. 37587561 10.1158/1078-0432.CCR-23-0547 PMC10502465

[12] Jacobsen PB, Jim HS. Psychosocial interventions for anxiety and depression in adult cancer patients: achievements and challenges. CA Cancer J Clin. 2008;58:214–30. 10.3322/CA.2008.0003. 18558664 10.3322/CA.2008.0003

[13] Miller K, Massie MJ. Depression and anxiety. Cancer J. 2006;12:388–97. 10.1097/00130404-200609000-00008. 17034675 10.1097/00130404-200609000-00008

[14] Stark DP, House A. Anxiety in cancer patients. Br J Cancer. 2000;83:1261–7. 10.1054/bjoc.2000.1405. 11044347 10.1054/bjoc.2000.1405 PMC2408796

[15] PD JE. Recent updates in the management of chemotherapy-induced nausea and vomiting. Asian J Pharm Clin Res. 2013;6(Suppl 1):5–10. https://journals.innovareacademics.in/index.php/ajpcr/article/view/323

[16] Tsilimigras DI, Woldesenbet S, Chatzipanagiotou OP, et al. Long-term lorazepam use may be associated with worse long-term outcomes among patients with pancreatic adenocarcinoma. Surgery. 2024. 10.1016/j.surg.2024.08.027. 39304446 10.1016/j.surg.2024.08.027

[17] Cornwell AC, Feigin ME. Unintended effects of GPCR-targeted drugs on the cancer phenotype. Trends Pharmacol Sci. 2020;41:1006–22. 10.1016/j.tips.2020.10.001. 33198923 10.1016/j.tips.2020.10.001 PMC7672258

[18] Bishop JF, Olver IN, Wolf MM, et al. Lorazepam: a randomized, double-blind, crossover study of a new antiemetic in patients receiving cytotoxic chemotherapy and prochlorperazine. J Clin Oncol. 1984;2:691–5. 10.1200/JCO.1984.2.6.691. 6374058 10.1200/JCO.1984.2.6.691

[19] James A, Nair MM, Abraham DS, et al. Effect of lorazepam in reducing psychological distress and anticipatory nausea and vomiting in patients undergoing chemotherapy. J Pharmacol Pharmacother. 2017;8:112. 10.4103/jpp.JPP_54_17. 29081618 10.4103/jpp.JPP_54_17 PMC5642123

[20] Xie HG, Kim RB, Wood AJ, Stein CM. Molecular basis of ethnic differences in drug disposition and response. Annu Rev Pharmacol Toxicol. 2001;41:815–50. 10.1146/annurev.pharmtox.41.1.815. 11264478 10.1146/annurev.pharmtox.41.1.815

[21] Check DK, Basch EM. Appropriate use of antiemetics to prevent chemotherapy-induced nausea and vomiting. JAMA Oncol. 2017;3:307–9. 10.1001/jamaoncol.2016.2616. 27631790 10.1001/jamaoncol.2016.2616 PMC5558447

