# Supplementary material for: Lorazepam and Survival in Asian Patients with Pancreatic Cancer: A Retrospective Cohort Study
Source: J Gastrointest Cancer. 2026 Mar 2;57(1):55. doi: 10.1007/s12029-026-01429-7 (PMC12953360; doi:10.1007/s12029-026-01429-7)
Supplement: Supplementary file 1 — Supplementary file1 (DOCX 53 KB) [file 12029_2026_1429_MOESM1_ESM.docx]

**Supplementary Tables.**

**Supplementary Table 1. Detailed chemotherapy regimens**

|  | Low-dose group  (N=383) | High-dose group  (N=226) |
| --- | --- | --- |
| Neo-Adjuvant therapy |  |  |
| None | 372 (97.1%) | 220 (97.3%) |
| CCRT only | 2 (0.5%) | 1 (0.4%) |
| CCRT + Adjuvant Gemcitabine | 1 (0.3%) | 0 (0.0%) |
| FOLFIRINOX | 7 (1.8%) | 4 (1.8%) |
| Gemcitabine/nab-paclitaxel | 1 (0.3%) | 0 (0.0%) |
| SABR | 0 (0.0%) | 1 (0.4%) |
| Adjuvant therapy |  |  |
| None | 319 (83.2%) | 204 (90.3%) |
| CCRT only | 14 (3.7%) | 7 (3.1%) |
| CCRT following adjuvant 5-FU | 9 (2.3%) | 3 (1.3%) |
| CCRT following adjuvant FL | 6 (1.6%) | 0 (0.0%) |
| CCRT following adjuvant Gemcitabine | 9 (2.3%) | 3 (1.3%) |
| FL | 5 (1.3%) | 2 (0.9%) |
| FOLFIRINOX | 1 (0.3%) | 2 (0.9%) |
| FOLFIRINOX following FL | 1 (0.3%) | 0 (0.0%) |
| Gemcitabine/nab-paclitaxel | 1 (0.3%) | 0 (0.0%) |
| Gemcitabine | 15 (3.9%) | 4 (1.8%) |
| Gemcitabine following CCRT | 1 (0.3%) | 1 (0.4%) |
| Gemcitabine following FL | 1 (0.3%) | 0 (0.0%) |
| Trial | 1 (0.3%) | 0 (0.0%) |
| 1st palliative chemotherapy |  |  |
| None | 0 (0.0%) | 0 (0.0%) |
| FOLFIRINOX | 243 (63.4%) | 164 (72.6%) |
| FOLFOX | 1 (0.3%) | 0 (0.0%) |
| Gemcitabine/nab-paclitaxel | 133 (34.7%) | 60 (26.5%) |
| Gemcitabine | 2 (0.5%) | 2 (0.9%) |
| Gemcitabine/Erlotinib | 1 (0.3%) | 0 (0.0%) |
| Gemcitabine/paclitaxel | 1 (0.3%) | 0 (0.0%) |
| Gemcitabine/Cisplatin | 2 (0.5%) | 0 (0.0%) |
| 2nd palliative chemotherapy |  |  |
| None | 128 (33.4%) | 62 (27.4%) |
| FL | 2 (0.5%) | 0 (0.0%) |
| FOLFIRI | 1 (0.3%) | 1 (0.4%) |
| FOLFIRINOX | 42 (11.0%) | 32 (14.2%) |
| FOLFOX | 17 (4.4%) | 11 (4.9%) |
| Gemcitabine/nab-paclitaxel | 71 (18.5%) | 77 (34.1%) |
| Gemcitabine | 25 (6.5%) | 8 (3.5%) |
| Gemcitabine/Erlotinib | 33 (8.6%) | 14 (6.2%) |
| Gemcitabine/Cisplatin | 15 (3.9%) | 5 (2.2%) |
| iFAM | 1 (0.3%) | 0 (0.0%) |
| NAPOLI | 3 (0.8%) | 2 (0.9%) |
| S-1 | 16 (4.2%) | 4 (1.8%) |
| Trial | 27 (7.0%) | 10 (4.4%) |
| Capecitabine/Oxaliplatin | 2 (0.5%) | 0 (0.0%) |
| 3rd palliative chemotherapy |  |  |
| None | 261 (68.1%) | 157 (69.5%) |
| FOLFIRI | 2 (0.5%) | 0 (0.0%) |
| FOLFIRINOX | 1 (0.3%) | 0 (0.0%) |
| FOLFOX | 2 (0.5%) | 2 (0.9%) |
| 5-FU/Cisplatin | 1 (0.3%) | 0 (0.0%) |
| Gemcitabine/nab-paclitaxel | 4 (1.0%) | 1 (0.4%) |
| Gemcitabine | 3 (0.8%) | 2 (0.9%) |
| Gemcitabine/Erlotinib | 5 (1.3%) | 3 (1.3%) |
| Gemcitabine/Cisplatin | 1 (0.3%) | 0 (0.0%) |
| iFAM | 7 (1.8%) | 5 (2.2%) |
| NAPOLI | 57 (14.9%) | 30 (13.3%) |
| S-1 | 34 (8.8%) | 23 (10.2%) |
| Trial | 3 (0.8%) | 3 (1.3%) |
| Capecitabine | 1 (0.3%) | 0 (0.0%) |
| Capecitabine/Oxaliplatin | 1 (0.3%) | 0 (0.0%) |
| 4th palliative chemotherapy |  |  |
| None | 336 (87.7%) | 204 (90.3%) |
| 5-FU/Cisplatin | 2 (0.5%) | 2 (0.9%) |
| Gemcitabine/nab-paclitaxel | 2 (0.5%) | 0 (0.0%) |
| Gemcitabine/Cisplatin | 1 (0.3%) | 0 (0.0%) |
| iFAM | 3 (0.8%) | 4 (1.8%) |
| IRIS | 1 (0.3%) | 0 (0.0%) |
| Pembrolizumab | 1 (0.3%) | 0 (0.0%) |
| NAPOLI | 8 (2.1%) | 7 (3.1%) |
| S-1 | 26 (6.8%) | 9 (4.0%) |
| Trial | 3 (0.8%) | 0 (0.0%) |
| 5th palliative chemotherapy |  |  |
| None | 374 (97.7%) | 224 (99.1%) |
| Doxorubicin/mitomycin-C | 1 (0.3%) | 0 (0.0%) |
| FOLFIRINOX | 1 (0.3%) | 0 (0.0%) |
| 5-FU/Cisplatin | 1 (0.3%) | 1 (0.4%) |
| NAPOLI | 2 (0.5%) | 0 (0.0%) |
| S-1 | 3 (0.8%) | 1 (0.4%) |
| Capecitabine | 1 (0.3%) | 0 (0.0%) |
| 6th palliative chemotherapy |  |  |
| None | 382 (99.7%) | 225 (99.6%) |
| Gemcitabine/nab-paclitaxel | 0 (0.0%) | 1 (0.4%) |
| Trial | 1 (0.3%) | 0 (0.0%) |
| 7th palliative chemotherapy |  |  |
| None | 383 (100.0%) | 225 (99.6%) |
| Capecitabine | 0 (0.0%) | 1 (0.4%) |

FL, 5-FU/Leucovorin; CCRT, Concurrent Chemoradiotherapy; FOLFIRI, 5-FU/Leucovorin + Irinotecan; FOLFIRINOX, 5-FU/Leucovorin + Irinotecan + Oxaliplatin; FOLFOX, 5-FU/Leucovorin + Oxaliplatin; iFAM, Ifosfamide + 5-Fluorouracil + Adriamycin (Doxorubicin) + Mitomycin-C; IRIS, Irinotecan + S-1; NAPOLI, Nanoliposomal Irinotecan + 5-FU/Leucovorin; S-1, Tegafur/Gimeracil/Oteracil; SABR, Stereotactic Ablative Body Radiotherapy.
